# A Systematic Review of Economic Evaluations in Clinical Nursing Practices

**DOI:** 10.1155/2024/9939254

**Published:** 2024-08-09

**Authors:** Yushan Guan, Nan Ru, Ruifu Kang, Xiangping Jia, Tingting Xu, Zhaolin Meng

**Affiliations:** ^1^ School of Nursing Capital Medical University, Beijing, China; ^2^ Department of Health Policy and Management Capital Medical University, Beijing, China

## Abstract

**Background:**

The misallocation of scarce healthcare resources globally raises concerns regarding the underuse of high-value care and the overuse of low-value care. Economic evaluations can help policy makers determine whether an intervention presents a better value for money and desirable clinical benefits, thus realizing value-based care.

**Aim:**

We aimed to conduct a systematic review of the economic evaluations of clinical nursing practices to advance knowledge on value-based care.

**Methods:**

A systematic review was conducted using MEDLINE, Embase, Web of Science, Cochrane Central Register of Controlled Trials, CINAHL, NHS Economic Evaluation Database, Health Technology Assessment, and Tufts CEA Registry for full economic evaluations of clinical nursing practices from January 2013 to January 2023. Outcomes were incremental cost-effectiveness ratios, incremental cost-utility ratios, incremental cost-benefit ratios, incremental net benefit, and the differences in costs for cost-minimization studies. Methodological quality was evaluated using the Consensus Health Economic Criteria–extended checklist. Results were synthesized using permutation matrices for all studies. The protocol was registered with PROSPERO (CRD42023415918).

**Results:**

Thirty-five studies were included in this review, with 27 studies categorized as good methodological quality and 8 as moderate quality. Clinical nursing practices were dominant (i.e., more effective and less costly) in 19 studies, potentially cost-effective depending on willingness-to-pay thresholds in 15 studies, and were dominated (i.e., less effective and more costly) in 1 study.

**Conclusion:**

Our study advanced knowledge on value-based care for clinical nursing practices. Results suggest that most clinical nursing practices studied may be clearly economically favourable or potentially favourable. *Implications for Nursing Management*. The results of this review provide valuable insights into value-based care in nursing and facilitate the decision-making of healthcare policymakers regarding health resource allocation to achieve value-based care.

## 1. Introduction

Since nursing care represents a significant proportion of healthcare expenditures, there are substantial opportunities for nurses to contribute to and lead efforts in cost reduction and healthcare improvement [1, 2]. Compared to the economic value of clinical practices provided by medical specialists, less attention is paid to the economic value of clinical nursing practices [2]. Clinical nursing practices were defined as clinical interventions, programs, or approaches to care that were delivered by or ordered by nurses, or led by nurses, within the scopes of registered nurse practice or advanced practice nursing [1]. The research that examines the economic value of clinical nursing practices can inform nurse decision-makers, seeking to allocate limited financial resources efficiently and effectively, thus eventually contributing to the realization of value-based care.

Value-based care is defined as a framework for optimizing health and well-being per unit of expenditure [3]. When patients do not receive care that is highly likely to improve the quality or quantity of life, which represents good value for money, the phenomenon is commonly referred to as underuse of high-value clinical practice. Conversely, when patients receive care in which evidence suggests its potential harm exceeds the potential benefit or the additional costs do not provide proportional added benefits, it is commonly referred to as overuse of low-value clinical practice [3, 4]. It has been estimated that approximately 30% of healthcare spending is on low-value clinical practice, which wastes limited resources and may threaten the sustainability of the healthcare system [2, 5].

To achieve value-based care, both the underuse of high-value clinical practice and the overuse of low-value clinical practice should be focused on, with supportive evidence from economic evaluations [6]. Economic evaluation is the comparative analysis of alternative courses of action in terms of both their costs and consequences, which can help determine whether clinical practices present a better value for money and desirable use of healthcare [7, 8]. There are four types of full economic evaluations: cost-effectiveness analysis (CEA), cost-utility analysis (CUA), cost-benefit analysis (CBA), and cost-minimization analysis (CMA), which differ in the way consequences are measured. CEA measures consequences in natural (health) units, such as life years gained, whereas CUA measures outcomes in a single unit of measurement, such as quality-adjusted life year (QALY). CBA measures consequences in monetary terms, which can directly express whether the benefits outweigh the costs. CMA is a method of comparing different alternatives that use comparisons of costs when benefits have shown to be equivalent [8].

Earlier efforts to summarize the evidence on economic evaluations of clinical nursing practices are limited. One previous review evaluated and described the quantity and quality of economic evaluations in nursing-relevant research conducted in the United States (USA) between 1997 and 2015 [1]. However, to the best of the authors' knowledge, no comprehensive systematic review of economic evaluations in the field of nursing has been conducted to identify both low- and high-value clinical nursing practices. Our study aimed to conduct a systematic review of the economic value of clinical nursing practices to advance knowledge of value-based care. The results of this review can facilitate the decision-making of healthcare policymakers regarding health resource allocation to achieve value-based care.

## 2. Methods

### 2.1. Design

We followed the guideline recommendations for conducting systematic reviews of economic evaluations for informing evidence-based healthcare decisions [9–11], and all findings were reported according to the updated version of the Preferred Reporting Items for Systematic Reviews and Meta-Analyses (PRISMA 2020) guidelines [12]. The protocol was registered with the International Prospective Register of Systematic Reviews (PROSPERO CRD42023415918).

### 2.2. Inclusion and Exclusion Criteria

We included full economic evaluations (cost-effectiveness, cost-utility, cost-benefit, and cost-minimization analyses) of clinical nursing practices. Both trial-based and model-based economic evaluations were included. Clinical nursing practices were defined as clinical interventions, programs, or approaches to care that were delivered by or ordered by nurses, or included nurses, within the scopes of registered nurse practice or advanced practice nursing [1]. We restricted the review to studies published in English from January 2013 to January 2023 to ensure its feasibility and timeliness of the results. In addition, we considered studies identifying the economic evaluation results wherein one clinical practice being dominant or dominated by the alternative.

We excluded studies of practices provided by other healthcare professionals or nursing procedures that require an order from other healthcare professionals such as medication prescribing or requests for lab testing by physicians. We also excluded research protocols, conference abstracts, editorials, letters to editor, and narrative reviews.

### 2.3. Search Strategy and Study Selection

An extensive literature search was conducted in MEDLINE (via Ovid), Embase, National Health Services Economic Evaluation Database (NHS EED), Health Technology Assessment (HTA) Database, Cochrane Central Register of Controlled Trials (via Ovid), Cumulative Index to Nursing and Allied Health Literature (CINAHL), Web of Science, and the Tufts Cost-Effectiveness Analysis Registry (https://www.cearegistry.org). The full search strategies for each database are presented in Appendix 1. Furthermore, reference lists of included studies and studies cited in previous reviews were also screened to identify additional studies.

All citations were exported to EndNote (version X8) in which the duplications were eliminated. A screening web tool system, RAYYAN (https://rayyan.ai/), was then used for the screening process. To ensure reliability, sets of 200 citations were independently evaluated and then discussed by the reviewers until acceptable agreement was achieved during study selection. Two reviewers (YSG and RFK) then independently screened all identified records using titles and abstracts. All abstracts that could potentially apply to the inclusion criteria were forwarded to full-text review. Any disagreement was resolved through discussion between reviewers and, if necessary, consultation with a third reviewer (ZLM).

### 2.4. Data Extraction

An electronic data extraction form was developed, with a detailed instruction manual and piloted on a representative sample of 10 publications. Pairs of reviewers (YSG and ZLM) independently extracted the following information from eligible studies: country, intervention and comparator, study design (single study-based and model-based), type of economic evaluation, population, perspective, threshold, cost categories, outcomes, and cost-effectiveness results (incremental cost-effectiveness ratio (ICER), incremental cost-utility ratio (ICUR), incremental cost-benefit ratio, incremental net benefit, and the difference in costs for cost-minimization studies). Any conflict was resolved through consensus. In cases of disagreement, a third reviewer (TTX) with content expertise in economic evaluation was consulted. The corresponding authors were contacted by emails to obtain additional information when necessary.

### 2.5. Quality of Methodology Assessment

Two reviewers independently assessed the methodological quality for each included study using the recommended approach, the Consensus Health Economic Criteria (CHEC)–extended checklist (Appendix 2) [13, 14]. This 20-item checklist can appraise model-based or trial-based studies, with positive responses scored 1 and negative responses scored 0 [10]. The total score for each item was summed and converted to a percentage with the final scores ranging from zero to 100 (final score = (total score/20) × 100%). The total CHEC score for each study was categorized into four grades based on cutoff values: low (≤50), moderate (51–75), good (76–95), and excellent (>95) [15]. Higher scores indicate higher quality. We also presented the results in a graph using RevMan 5.3 software.

### 2.6. Data Synthesis and Analysis

Meta-analyses were not performed due to the heterogeneity of the costing methods and the interventions, in line with Cochrane guidelines [16]. Instead, a narrative synthesis of the findings from included studies was presented. We used a permutation matrix summarizing results according to nine possible outcomes of cost (higher, same, and lower) and effectiveness (higher, same, and lower), which was adapted from the systematic review of economic evaluation guidelines developed by Joanna Briggs Institute [17]. Costs were converted to 2024 international dollars (Int.$) using a web-based tool [18]. For studies that did not report the reference year, an assumption of base year prior to the publication date was made as the base year. For cost-utility studies, we used a willingness-to-pay threshold of $50 000 per QALY gained to assist in interpreting the results [19]. Our goal was to be as broad as possible to assist the readership with the interpretation of results in line with their settings so we did not consider applicability or transferability to specific settings in the synthesis methods.

## 3. Results

### 3.1. Results of the Search

**Figure 1:**
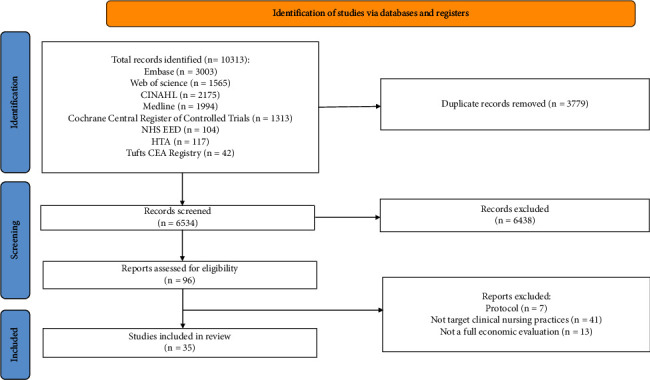
PRISMA flowchart of the study selection process. Note: PRISMA indicates Preferred Reporting Items for Systematic Reviews and Meta-Analyses.

The initial search identified 10,313 studies. After excluding duplicate studies, 6,534 records remained for title and abstract screening. Of these, 96 were eligible for full-text assessment. After the full-text screening, 35 studies were included in the systematic review. The study selection process is summarized in Figure 1. Sixty-one full-text articles were excluded because they did not meet the “full economic evaluation” criteria (*n*=13), were not primary studies reporting results of an economic evaluation (e.g., protocols) (*n*=7), or did not target clinical nursing practices (*n*=41) (see Appendix 3).

### 3.2. Characteristics of Included Studies

The characteristics of included studies are shown in Table 1. Among the 35 included articles, 28 studies were conducted in high-income countries: the United Kingdom (*n*=6), Australia (*n*=5), the United States (*n*=5), Canada (*n*=2), Germany (*n*=2), Sweden (*n*=1), Denmark (*n*=1), the Netherlands (*n*=1), Italy (*n*=1), Ireland (*n*=1), Japan (*n*=1), Korea (*n*=1), and multicountry (*n*=1). Seven studies were conducted in low- and middle-income countries (LMICs): China (*n*=3), Brazil (*n*=3), and Turkey (*n*=1). Eighteen studies were published between 2018 and 2022 and 17 studies between 2013 and 2017. A total of 16 studies were single study-based economic evaluations and 19 were model-based economic evaluations. Most studies (*n*=23) performed a cost-effectiveness analysis, 15 performed a cost-utility analysis, and four performed a cost-benefit analysis (five of these performed both a cost-effectiveness and a cost-utility analysis; two of these performed both a cost-effectiveness and a cost-benefit analysis). The majority of studies were conducted in hospital settings (*n*=29), while six studies were conducted in primary care settings. Sixteen economic evaluations were conducted from a healthcare system perspective, eight from a societal perspective, and five from a payer perspective (two of which were conducted from both a healthcare system and societal perspective). The perspective was not reported in eight studies. A total of 18 economic evaluation studies assessed the cost-effectiveness of treatment practices, and 17 studies assessed prevention practices. Targeted economic evaluations of treatment practices included wound care (*n*=4) [20–23], urinary catheterisation care (*n*=1) [24], intravenous therapy (*n*=3) [25–27], medication administration (*n*=1) [28], pressure ulcer management (*n*=1) [29], peristomal skin care (*n*=1) [30], oxygen therapy (*n*=1) [31], pulse oximetry monitoring (*n*=1) [32], nutrition management (*n*=1) [33], nasogastric tube placement (*n*=1) [34], immobile patient care (*n*=1) [35], respiratory rate monitoring (*n*=1) [36], and doula care (*n*=1) [37]. Targeted economic evaluations of prevention practices included pressure ulcer prevention (*n*=9) [38–46], catheter-associated urinary tract infection prevention (*n*=2) [47, 48], central venous catheter-related infection prevention (*n*=1) [49], medication error prevention (*n*=1) [50], fall prevention (*n*=2) [51, 52], delirium prevention (*n*=1) [53], and aspiration prevention (*n*=1) [54].

### 3.3. Methodological Quality of Included Studies

Table 2 and Appendix 4 summarize the results of the risk of bias assessment of the included studies, as indicated by the percentage score. Each item was scored as 1 if the study met the requirement or 0 if it did not meet or only partially met the requirement. Moreover, “not applicable” (NA) was noted and was scored as 1 if the assessed item was not relevant to the study. The quality of all studies ranged from 60% to 95%. A total of 27 studies [20, 21, 23, 25–32, 34–38, 41, 42, 44–49, 51, 52, 54] were categorized as good quality, eight [22, 24, 33, 39, 40, 43, 50, 53] as moderate quality, and no study as low quality. Two items, model description (item 5) and generalizability (item 18), had the lowest scores. Of the 19 model-based studies, 13 (68.4%) did not report the structural assumptions and the validation methods of the model. Regarding generalizability, 21 studies (60.0%) did not discuss the generalizability of their results to other settings and populations. Other methodological shortcomings included the following: 12 studies (34.2%) did not state the ethical issue (item 20), nine studies (25.7%) did not identify all relevant costs (item 8), nine studies (25.7%) did not describe the population characteristics in detail (item 1), eight studies (22.9%) did not state the study perspective (item 7), and seven studies (20.0%) did not perform uncertainty analyses (item 16).

### 3.4. Results of Economic Evaluations

**Figure 2:**
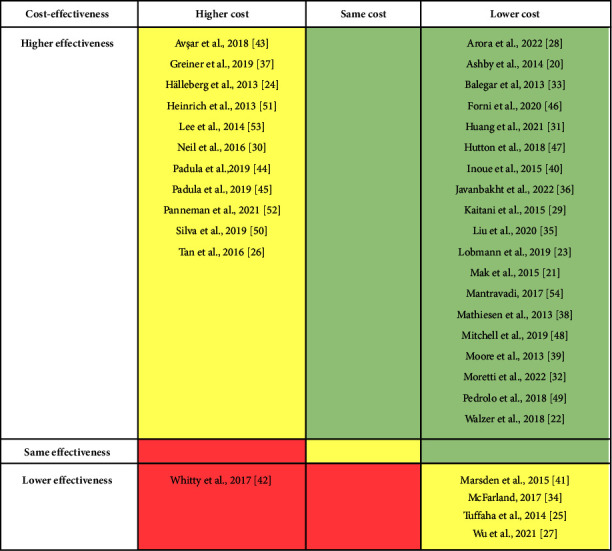
Permutation matrix. Note: areas highlighted in green indicate that interventions were dominant (i.e., at least as effective as lower cost). Areas highlighted in yellow indicate that interventions were potentially cost-effective at specified willingness-to-pay thresholds (i.e., more effective and more costly, or less effective and less costly). Areas highlighted in red indicate that interventions were dominated (i.e., the same or less effective and higher costs).

The results of the economic evaluations are presented in Table 3, Figure 2, and Appendix 5. Out of 35 economic evaluations, the intervention group was dominant (more effective and less costly when compared with the control) in 19 studies (Table 3, Figure 2, and Appendix 5). Five dominant interventions were identified from randomized controlled trials (RCTs) (see Appendix 6).

The intervention group was more effective and more costly in 11 studies, and the intervention was less effective and less costly in four studies (Table 3 and Figure 2). These interventions were likely to be cost-effective, at specified willingness-to-pay thresholds (Appendix 5). Among the six cost-utility studies that reported ICUR, the interventions were favoured in five of these [27, 34, 37, 44, 45] at a willingness-to-pay threshold of $50 000 per QALY gained.

As shown in Table 3, Figure 2, and Appendix 5, the intervention group was dominated (i.e., less effective and more costly) by the control in an economic evaluation alongside an RCT: a pressure ulcer prevention care bundle consisting of information and education resources targeted to patients (DVD, poster, brochure, and face-to-face education) and nurse training package was dominated by standard care which was aligned with regional guidelines [42].

## 4. Discussion

To the best of our knowledge, this is the first study to systematically review the scientific literature to summarize evidence on the economic value of clinical nursing practices. We identified 35 full economic evaluations published between January 2013 and January 2023. The clinical nursing practices were clearly economically favourable (more effective with lower costs) in 19 out of 35 economic evaluations (54.3%), potentially favourable (more effective with higher costs or less effective with lower costs) in 15 (42.9%), and unfavourable (less effective and more costly) in 1 (2.9%).

It was noteworthy that most interventions were clearly economically favourable or potentially favourable, while only one intervention about pressure ulcer prevention care bundle might represent low-value care (less effective and more costly). Of the economic evaluations included, the number of cost-effective interventions was significantly greater than that of the non-cost-effective. While multifactorial, part of the reason may be that the lack of standardization of the types of costs included and the lack of evaluations from the societal perspective, which may result in misestimating the value of the intervention. In most of the economic evaluation studies included, which were conducted from a healthcare system perspective, only direct costs of the interventions were considered, without including other indirect costs such as productivity costs. Moreover, there may be a risk of publication bias, representing a small fraction of the nursing interventions evaluated for effectiveness, even among those found to be effective [55]. This probably led to misestimates of the economic benefit of the interventions. The findings suggest the importance of emphasizing the publication of negative studies as much as the positive studies, as the absence of either may lead to publication bias.

It was also of interest to note that five interventions were clearly dominant compared to their comparators, supported by evidence from RCTs. In the hierarchy of research designs, the results of RCTs are considered the highest level of evidence, whereas observational studies are susceptible to potential confounding biases [56]. Therefore, these comparators, with a high level of evidence (RCTs), may present opportunities to reduce low-value care through deimplementation. For instance, “do not recommend routinely repositioning 6 h, using the 90° lateral rotation as the first repositing choice for most patients to prevent the pressure ulcers” [39], “do not recommend four-layer bandage for healing of venous leg ulcers as the first choice” [20], “do not recommend routinely continuous pulse oximetry monitoring for the supportive care of hospitalized infants with bronchiolitis” [32], “do not recommend nasal high-flow oxygen for treating respiratory distress in newborns as sole primary support” [31], and “do not recommend swabbing methods in cleansing wounds healed by secondary intention as the first choice, instead, pressurized irrigation” [21].

It should also be considered that although this review included all international articles meeting the inclusion criteria, only 35 full economic evaluations of clinical nursing practices were identified. Moreover, the majority of the economic evaluation studies were conducted in high-income countries, mostly in the UK, the USA, or Australia, with limited evidence from LMICs. In LMICs, where healthcare resources are scarce, more attention should be paid to the evidence from economic evaluation studies to aid in decision-making regarding the allocation of limited healthcare resources. Moreover, studies conducted in LMICs examining the economic evaluations of clinical nursing practices would increase the generalizability of the findings.

In terms of methodological quality, all the included studies demonstrated moderate to good quality, but some methodological limitations were observed. For example, a significant portion of model-based studies (68.4%) failed to specify the structural assumptions or the validation methods of the model. Also, many studies (60.0%) [20, 22–24, 27, 29, 30, 34–36, 38–43, 48, 49, 51, 53, 54] did not discuss the generalizability of the results. In addition, some studies (22.9%) [21, 24, 26, 33, 39, 40, 50, 53] did not specify the perspective adopted in the study, which is critical for the identification of cost components. Moreover, some studies (20%) [24–26, 33, 39, 40, 43] did not incorporate uncertainty analyses. Ideally, both deterministic and probabilistic uncertainty analyses should be conducted within a single economic evaluation to reflect the uncertainty of parameters [57]. These analyses are helpful for assessing the reliability of cost-effectiveness inferences and informing the direction of further research [57]. These findings implied that more attention should be paid to the methodological rigor in these items of economic evaluations of clinical nursing practices in the future.

### 4.1. Strengths and Limitations

To our knowledge, this is the first systematic review on full economic evaluations of clinical nursing practices, addressing a major knowledge gap on the economic value of clinical nursing practices in both developing and developed countries. We used a comprehensive search strategy that encompassed multiple electronic databases to identify relevant economic evaluations. In addition, two independent reviewers conducted a rigorous quality assessment of the included studies using the CHEC-extended list.

However, our review has several limitations. Although we used a comprehensive search strategy and searched specialised databases, we only searched electronic databases, potentially overlooking economic evaluations of clinical nursing practices that were not available in these databases. Moreover, we restricted the review to studies published in English since January 2013 to ensure its feasibility and relevance to current practices in value-based care. In addition, as cost-effectiveness measures cannot generally be compared across studies, quantitative synthesis was not advisable. Instead, we synthesized results qualitatively using a permutation matrix. We attempted to facilitate comparison between cost-utility studies by applying a unique willingness-to-pay threshold, while the results obtained will be dependent on the chosen threshold. In our study, the cost-utility studies indicating potentially favourable interventions based on willingness-to-pay thresholds were all conducted in high-income countries, where the threshold ($50 000 per QALY gained) used in our study is widely applied in the literature. Furthermore, the heterogeneity in cost estimates, which may not be generalizable across different regions or countries, is another limitation of our study.

## 5. Conclusions

Despite these limitations, our study systematically reviewed the evidence regarding the economic value of clinical nursing practices to advance knowledge on value-based care. We found that interventions were dominant in 19 studies, likely cost-effective in 15 studies, and dominated by comparators in 1 study. Results of this review can help nurses and decision-makers to assess the value of clinical nursing practices. However, caution is needed in extrapolating the results of our study, given the potential for publication bias and certain methodological limitations in the reviewed studies. More high-quality economic evaluations in clinical nursing practices are warranted to provide evidence of their value, particularly in LMICs, improving the cost-effectiveness of healthcare delivery and facilitating the realization of value-based care.

## Figures and Tables

**Table 1:** Overview of included studies (*N*=35).

Characteristics	*n* (%)
*Country*	
UK	6 (17.1)
Australia	5 (14.3)
USA	5 (14.3)
China	3 (8.6)
Brazil	3 (8.6)
Canada	2 (5.7)
Germany	2 (5.7)
Multicountry (USA and Australia)	1 (2.8)
Others	8 (22.9)

*Year of publication*	
2013–2017	17 (48.6)
2018–2022	18 (51.4)

*Study design*	
Single study-based	16 (45.7)
Model-based	19 (54.3)

*Types of economic evaluation*	
Cost-effectiveness analysis	16 (45.7)
Cost-utility analysis	10 (28.6)
Cost-benefit analysis	2 (5.7)
Cost-utility and cost-effectiveness analyses	5 (14.3)
Cost-effectiveness and cost-benefit analyses	2 (5.7)

*Setting of economic evaluation*	
Hospital setting	29 (82.9)
Primary care setting	6 (17.1)

*Perspective*	
Healthcare system	14 (40.0)
Societal	6 (17.1)
Payer	5 (14.3)
Societal and healthcare system	2 (5.7)
Not stated	8 (22.9)

*Types of clinical nursing practices*	
Treatment practices	18 (51.4)
Prevention practices	17 (48.6)

**Table 2:** Quality assessment of included studies using CHEC-extended checklist.

First author, year	1	2	3	4	5	6	7	8	9	10	11	12	13	14	15	16	17	18	19	20	Score (%)	Grade
Arora et al., 2022 [28]	0	1	1	1	1	1	1	1	1	1	1	1	1	1	1	1	1	1	1	1	95	Good
Ashby et al., 2014 [20]	1	1	1	1	NA (1)	1	1	1	1	1	1	1	1	1	1	1	1	0	1	1	95	Good
Avşar et al., 2018 [43]	1	1	1	1	NA (1)	0	1	1	1	0	1	1	0	0	1	0	1	0	0	0	60	Moderate
Balegar et al., 2013 [33]	1	1	1	1	NA (1)	1	0	0	0	0	1	1	1	0	0	0	1	1	1	1	65	Moderate
Forni et al., 2020 [46]	1	1	1	1	0	1	1	1	1	0	0	1	1	1	1	1	1	1	1	0	80	Good
Greiner et al., 2019 [37]	1	1	1	1	0	1	1	1	1	1	1	1	1	1	1	1	1	1	1	1	95	Good
Hälleberg et al., 2013 [24]	1	1	1	1	NA (1)	0	0	1	1	1	1	1	1	1	0	0	1	0	1	1	75	Moderate
Heinrich et al., 2013 [51]	1	1	1	1	NA (1)	1	1	1	1	1	1	1	1	1	1	1	1	0	1	1	95	Good
Huang et al., 2021 [31]	1	1	1	1	NA (1)	1	1	1	1	1	1	1	1	0	1	1	1	1	1	0	90	Good
Hutton et al., 2018 [47]	0	1	1	1	0	1	1	1	1	1	1	1	1	1	1	1	1	1	1	1	90	Good
Inoue et al., 2015 [40]	1	1	1	1	NA (1)	1	0	0	1	1	1	1	1	1	1	0	1	0	1	0	75	Moderate
Javanbakht et al., 2022 [36]	1	1	1	1	0	1	1	1	1	1	1	1	0	1	1	1	1	0	1	1	85	Good
Kaitani et al., 2015 [29]	1	1	1	1	1	1	1	1	1	1	1	1	1	1	1	1	1	0	1	0	90	Good
Lee et al., 2014 [53]	0	1	1	1	NA (1)	1	0	1	1	1	1	1	1	1	1	1	1	0	0	0	75	Moderate
Liu et al., 2020 [35]	1	0	1	1	NA (1)	1	1	0	1	1	1	1	1	1	1	1	1	0	1	1	85	Good
Lobmann et al., 2019 [23]	1	1	1	1	0	1	1	1	1	1	1	1	1	1	1	1	1	0	1	1	90	Good
Mak et al., 2015 [21]	1	1	1	1	NA (1)	1	0	1	1	1	1	1	1	0	1	1	1	1	1	0	85	Good
Mantravadi, 2017 [54]	1	1	1	1	0	1	1	1	1	1	1	1	1	1	1	1	1	0	0	0	80	Good
Marsden et al., 2015 [41]	1	1	1	1	1	1	1	1	1	1	1	1	1	1	1	1	1	0	1	1	95	Good
Mathiesen et al., 2013 [38]	0	1	1	1	0	1	1	1	1	1	1	1	1	1	1	1	1	0	0	1	80	Good
McFarland, 2017 [34]	1	1	1	1	1	1	1	1	1	1	1	1	1	1	1	1	1	0	1	1	95	Good
Mitchell et al., 2019 [48]	1	1	1	1	0	1	1	0	1	1	1	1	1	0	1	1	1	0	1	1	80	Good
Moore et al., 2013 [39]	0	1	1	1	NA (1)	0	0	0	1	1	1	1	1	1	1	0	1	0	1	1	70	Moderate
Moretti et al., 2022 [32]	1	1	1	1	NA (1)	0	1	1	1	1	1	1	1	1	1	1	1	1	1	1	95	Good
Neil et al., 2016 [30]	1	1	1	1	1	1	1	0	1	1	1	1	1	0	1	1	1	0	1	0	80	Good
Padula et al., 2019 [44]	1	1	1	1	0	1	1	0	1	1	1	1	1	1	1	1	1	1	1	0	85	Good
Padula et al., 2019 [45]	0	1	1	1	0	1	1	1	1	1	1	1	1	1	1	1	1	1	1	1	90	Good
Panneman et al., 2021 [52]	0	1	1	1	0	1	1	1	1	1	1	1	1	0	0	1	1	1	1	1	80	Good
Pedrolo et al., 2018 [49]	1	1	1	1	1	1	1	0	1	1	0	1	1	1	1	1	1	0	1	1	85	Good
Silva et al., 2019 [50]	1	1	1	1	0	0	0	1	1	1	1	1	1	1	1	1	1	1	0	0	75	Moderate
Tan et al., 2016 [26]	1	1	1	1	NA (1)	1	0	1	1	1	1	1	1	1	1	0	1	1	1	1	90	Good
Tuffaha et al., 2014 [25]	0	1	1	1	NA (1)	1	1	1	1	1	1	1	1	1	1	0	1	1	1	1	90	Good
Walzer et al., 2018 [22]	0	1	1	1	0	1	1	0	1	1	1	1	0	1	1	1	1	0	0	0	65	Moderate
Whitty et al., 2017 [42]	1	1	0	1	NA (1)	1	1	1	1	1	1	1	1	1	1	1	1	0	1	1	90	Good
Wu et al., 2021 [27]	1	1	1	1	NA (1)	1	1	1	1	1	1	1	1	1	1	1	1	0	1	1	95	Good

*Note*. NA: not applicable. The items 1–20 assessed in the Consensus Health Economic Criteria (CHEC)–extended checklist are shown in Appendix 2.

**Table 3:** Results of economic evaluations of clinical nursing practices.

First author, year	Country	Intervention and comparator	Study design	Evaluation type	Population	Perspective	Currency (price year)	Cost categories	Outcomes	Findings (costs in 2024 int.$)
*Lower cost and higher effectiveness*										

Arora et al., 2022 [28]	USA	Intervention: ready-to-administer syringe administration of intravenous opioids Control: traditional vial-and-syringe administration of intravenous opioids	Model	CEA	Inpatients	Healthcare system perspective	USD (2021)	Costs of drug preparation and administration, drug waste, and errors	Errors of medication preparation and administration	Cost Intervention: $8216.97 (Int.$9322.40) Control: $8399.58 (Int.$9529.58) Errors Intervention: 0.0005 Control: 0.0086 ICER: −$22 554 (–Int.$25 588.20) (dominant)

Ashby et al., 2014 [20]	UK	Intervention: two-layer hosiery Control: four-layer bandage	RCT	CUA	Adults with venous leg ulcer	Societal perspective	GBP (not stated, assumed 2012)	Costs of trial compression treatments and healthcare consultations	QALY	Cost Intervention: £1492.9 (Int.$2953.97) Control: £1795.3 (Int.$3552.32) QALY Intervention: 0.685 Control: 0.651 ICUR: dominant

Balegar et al., 2013 [33]	Australia	Intervention: 48 h-TPN bags Control: 24 h-TPN bags	Before-after study	CEA	Infants receiving TPN	Not stated	AUD (not stated, assumed 2011)	TPN-related expenses (expenses towards purchasing and dispensing)	CLABSI	Cost Intervention: AUD190 153.00 (Int.$171 093.51) Control: AUD 287 756.00 (Int.$258 913.53) CLABSI (per 1000-line days) Intervention: 0.4 Control: 0.8 ICER: dominant

Forni et al., 2020 [46]	Italy	Intervention: standard prevention and foam dressing Control: standard prevention only	Model	CEA	Older patients with hip fractures	Healthcare system perspective	Euros (2017) and USD (2017)	Costs of dressing and other materials, nursing time, and treating pressure ulcers	Incidence of pressure ulcer	Cost Italy Intervention: €327.63 (Int.$540.64) Control: €1059.27 (Int.$1747.95) US Intervention: $394.54 (Int.$493.92) Control: $1234.16 (Int.$1545.03) Incidence of pressure ulcer Intervention: 4.5% Control: 15.4% ICER: dominant

Huang et al., 2021 [31]	Australia	Intervention: continuous positive airway pressure (CPAP) Control: nasal high-flow (nHF)	RCT	CEA	Infants with respiratory distress	Healthcare system perspective	AUD (2019)	Inpatient costs at nontertiary special care nurseries and tertiary NICU and costs of interhospital transfers	Intubation and NICU transfer rates	Cost Intervention: AUD20 606 (Int.$16 747.10) Control: AUD 21 615 (Int.$17 567.14) Intubation rate Intervention: 5.9% Control: 13.9% NICU transfer rate Intervention: 9.2% Control:15.7% ICER: dominant

Hutton et al., 2018 [47]	USA	Intervention: catheter-associated urinary tract infection prevention program Control: standard care	Model	CUA	Patients in nursing homes	Healthcare system perspective	USD (2015)	Intervention and disease costs	QALY	Cost Intervention: $139 948 (Int.$180 317.17) Control: $173 986 (Int.$224 173.72) QALY lost from CAUTI Intervention: 0.35 Control: 0.55 ICUR: dominant

Inoue et al., 2015 [40]	Brazil	Intervention: transparent film dressing Control: hydrocolloid dressing	Cohort study	CEA	Adults with motor and/or neurological limitation for active mobilization in bed	Not stated	BRL (not stated, assumed 2013)	Cost of product	Proportion of patients without pressure ulcer	Cost Intervention: R$347.60 (Int.$298.35) Control: R$1904.00 (Int.$1634.24) Proportion of patients without pressure ulcer Intervention: 80% Control: 70% ICER: dominant

Javanbakht et al., 2022 [36]	UK	Intervention: automatic respiratory rate monitoring plus intermittent nurse-led respiratory rate monitoring Control: intermittent nurse-led respiratory rate monitoring	Model	CUA	Patients with pneumonia	Societal perspective	GBP (2019)	Costs of the intervention, inpatient care, and management of respiratory compromise	QALY	Cost Intervention: £4752.0 (Int.$8374.68) Control: £4973.4 (Int.$8764.86) QALY Intervention: 6.926 Control: 6.917 ICUR: dominant

Kaitani et al., 2015 [29]	Japan	Intervention: advanced pressure ulcer management protocol Control: conventional care	Model	CUA	Older patients with pressure ulcers	Healthcare system perspective	JPY (2015)	Treatment and labor costs	QALY	Model 1 Cost Intervention: JPY¥35 217 (Int.$423.94) Control: JPY¥67 907 (Int.$817.46) QALY Intervention: 0.77 Control: 0.74 ICUR: dominant Model 2 Cost Intervention: JPY¥130 567 (Int.$1571.75) Control: JPY¥256 068 (Int.$3082.51) QALY Intervention: 0.66 Control: 0.59 ICUR: dominant

Liu et al., 2020 [35]	China	Intervention: a nursing intervention program Control: routine care	Before-after study	CUA	Immobile patients with stroke	Healthcare system perspective	CNY (2016)	Cost of hospitalization	QALY	Costs Intervention: ¥46 921 (Int.$15 646.18) Control: ¥51 610 (Int.$17 209.77) QALY Intervention: 0.179 Control: 0.170 ICUR: dominant

Lobmann et al., 2019 [23]	Germany	Intervention: a TLC-sucrose octa sulfate (TLC-NOSF) dressing Control: a neutral dressing	Model	CEA	Patients with diabetic foot ulcers	Payer's perspective	Euros (not stated, assumed 2017)	Costs of nursing, medical consultations, wound care products, inpatient stays, and pharmacotherapy	Wound healing rate	20-week period Cost Intervention: €2864.21 (Int.$44643.75) Control: €2958.69 (Int.$4796.93) Wound-healing rate Intervention: 48% Control: 30% ICER: −€530.80 (–Int.$860.59) (dominant) 100-week period Cost Intervention: €5882.87 (Int.$9537.91) Control: €8449.39 (Int.$13 699.01) Wound-healing rate Intervention: 94% Control: 81% ICER: dominant

Mak et al., 2015 [21]	China	Intervention: pressurised irrigation Control: swabbing method	RCT	CEA	Patients with wounds healing by secondary intention	Not stated	HKD (not stated, assumed 2013)	Costs of wound cleansing materials, dressing fixation materials, supplementary dressing materials, and nurse labor	Time-to-wound healing	Cost Intervention: HK$243.7 (Int.$52.72) Control: HK$353.8 (int.$76.53)
										Time-to-wound healing Intervention: 11.4days Control: 14.5days ICER: dominant

Mantravadi, 2017 [54]	USA	Intervention: aspiration risk-reduction intervention Control: usual care	Model	CEA	Elderly cancer survivors	Societal perspective	USD (not stated, assumed 2015)	Costs of training, materials, and productivity	Number of aspirations averted	Cost Intervention: $7774.35 (Int.$10 016.93) Control: $11 425.70 (Int.$14 721.54) Number of aspirations averted Intervention: 43.78 Control: 37.38 ICER: dominant

Mathiesen et al., 2013 [38]	Denmark	Intervention: pressure ulcer bundle Control: standard care	Model	CEA	Inpatients	Healthcare system perspective	Euros (2011)	Costs of prevention, healing, complications, and nurses' time	Number of prevented pressure ulcers	Cost Intervention: €79.83 (Int.$102.99) Control: €118.45 (Int.$152.82) Number of prevented pressure ulcers Intervention: 90.7% Control: 81.4% ICER: dominant

Mitchell et al., 2019 [48]	Australia	Intervention: chlorhexidine for meatal cleaning prior to urinary catheter insertion Control: saline for meatal cleaning prior to urinary catheter insertion	Model	CEA/CUA	Inpatients who received a urinary catheter	Societal perspective	AUD (not stated, assumed 2017)	Costs of saline and chlorhexidine and antimicrobial therapy	Number of CAUTI prevented, number of asymptomatic bacteriuria prevented, and QALY	Incremental cost: −AUD387 909 (–Int.$332 553.12) Incremental number of CAUTI prevented: 69.96 Incremental number asymptomatic bacteriuria prevented: 2450.60 Incremental QALY: 1.43 ICER: dominant

Moore et al., 2013 [39]	Ireland	Intervention: repositioned every 3 hours, using the 30° tilt Control: repositioned every 6 hours, using the 90° lateral rotation	RCT	CEA	Older hospitalized patients	Not stated	Euros (not stated, assumed 2011)	Nurse time costs	Incidence of pressure ulcers	Cost Intervention: €206.6 (Int.$346.93) Control: €253.1 (Int.$425.01) Incidence of pressure ulcers Intervention: 3% Control: 11% ICER: dominant

Moretti et al., 2022 [32]	Canada	Intervention: intermittent pulse oximetry monitoring Control: continuous pulse oximetry monitoring	RCT	CEA	Hospitalized infants with stabilized bronchiolitis	Societal and healthcare system perspective	CAD (2020)	Costs of hospital admission, physician visits, returning to emergency department and any hospital readmissions, productivity lost, and childcare or caregiving	Length of hospital stays in hours	Cost Societal perspective Intervention: CAD6879 (Int.$6849.14) Control: CAD7428 (Int.$7395.76) Healthcare system perspective Intervention: CAD4195 (Int.$4176.79) Control: CAD4716 (Int.$4695.53) Length of stay Intervention: 37.4 hours Control: 38.5 hours ICER: dominant

Pedrolo et al., 2018 [49]	Brazil	Intervention: chlorhexidine-impregnated dressings Control1: gauze and medical tape for short-term central venous catheter Control2: transparent semipermeable	Model	CEA	Critically ill patients	Healthcare system perspective	USD (2017)	Costs of treatment, hospitalization, cultures for diagnosis and control, and replacement cost of infected catheter	Catheter-related infection prevented	Cost Intervention: $655 (Int.$819.99) Control1: $696 (Int.$871.32) Control2: $670 (Int.$838.77) Catheter-related infection prevented Intervention: 99% Control1: 96% Control2: 97% ICER: dominant

Walzer et al., 2018 [22]	UK	Intervention: HRTD Control: SAP, SADM, SAKM, and SAE	Model	CUA/CEA	Patients with venous leg ulcers	Payer's perspective	GBP (2017)	Inpatient cost and outpatient cost	Healing of the wound, QALY	Intervention vs. SAP Incremental cost: £37.60 (Int.$68.84) Incremental effect (healed): 0.92years Incremental QALY: 0.0017 ICER: −£40.75 (−Int.$74.61) ICUR: −£22 073.30 (−Int.$40 413.11) Intervention vs. SADM Incremental cost: £171.68 (Int.$314.32) Incremental effect (healed): 2.42years Incremental QALY: 0.0045 ICER: −£70.90 (−Int.$129.81) ICUR: −£38 403.23 (−Int.$70 310.91) Intervention vs. SAKM Incremental cost: £3.13 (Int.$5.73) Incremental effect (healed): 0.14years Incremental utilities: 0.0003 ICER: −£22.28 (–Int.$40.79) ICUR: −£12 065.77 (–Int.$22 090.73) Intervention vs. SAE Incremental cost: £43.63 (Int.$79.88) Incremental effect (healed): 0.92 years Incremental QALY: 0.0017 ICER: −£47.28 (–Int.$86.56) ICUR: −£25 612.37 (–Int.$46 892.65) ICER/ICUR: dominant

*Higher cost and higher effectiveness*										

Avşar et al., 2018 [43]	Turkey	Intervention: evidence-based nursing interventions to maintain tissue integrity Control: routine nursing care	Before-after study	CEA	Adult patients	Healthcare system perspective	USD (not stated, assumed 2016)	Costs of prevention and treatment	Deterioration of tissue integrity	Cost Intervention: $405.97 (Int.$517.89) Control: $328.99 (Int.$419.68) Deterioration of tissue integrity Intervention: 18.2% Control: 54.5% ICER: not reported

Greiner et al., 2019 [37]	USA	Intervention: doula care Control: no doula care	Model	CUA/CEA	Pregnant women	Societal perspectives	USD (2018)	Costs of birth, labor time, and treating complications	QALY	Cost (in millions) Intervention: $31 949 (Int.$39 057.82) Control: $31 764 (Int.$38 831.66) QALY Intervention: 41 917 334 Control: 41 909 717 ICUR: 24 287.78 (Int.$29 691.94)

Heinrich et al., 2013 [51]	Germany	Intervention: multifactorial fall prevention program Control: usual care	Non-RCT	CEA	Nursing home residents	Payer's perspective	Euros (not stated, assumed 2011)	Costs of inpatient care, nursing home care, intervention, informal care, and ambulatory	Time free of femoral fracture	Cost Intervention: €271.18 (Int.$485.56) Control: €239.13 (Int.$428.17) Time free of femoral fracture Intervention: 316.83 days Control: 315.40 days ICER: €7481 (Int.$13 394.99)

Lee et al., 2014 [53]	Korea	Intervention: delirium prevention strategy Control: usual care	Cohort study	CBA	Patients after liver transplantation surgery	Not stated	USD (2007)	Costs of consultation, medication, equipment, and additional nursing time	Incremental net benefit, benefit-cost ratio	Cost: $38.4 (Int.$55.91) Benefit: $5578 (Int.$8121.76) Incremental net benefit: $5539.6 (Int.$8065.85) Benefit-cost ratio: 145.3

Neil et al., 2016 [30]	Canada	Intervention: peristomal skin complications management with ostomy components Control: peristomal skin complications management without components	Model	CUA/CEA	Patients living with an ostomy	Payer's perspective	CAD (2014)	Costs of barriers/pouches, ostomy components, and clinical utilization	Peristomal skin complications events and quality-adjusted life day (QALD)	Cost Intervention: CAD2339 (Int.$2477.20) Control: CAD2200 (Int.$2329.99) Peristomal skin complications events Intervention: 520 Control: 650 QALD Intervention: 270538 Control: 269495 ICER: not reported

Padula et al., 2019 [44]	USA and Australia	Intervention: prevention protocol to prevent pressure injuries Control: standard prevention	Model	CUA	Older patients	Societal perspectives	USD (2017)	Costs of skin care management, of support surfaces, nursing, dressing, and material and labor	QALY	United States' perspective Cost Intervention: $211 695.96 (Int.$265 020.26) Control: $211 116.51 (Int.$264 294.85) QALY Intervention: 0.7805 Control: 0.7647 ICUR: $36 652.23 (Int.$45 884.60) Australia's perspective Cost Intervention: $59 410.67 (Int.$74 375.68) Control: $58 496.99 (Int.$73 231.85) QALY Intervention: 0.8449 Control: 0.7874 ICUR: $15 898.83 (Int.$19 903.60)

Padula et al., 2019 [45]	USA	Intervention 1: repeated risk-assessment for pressure-injury prevention in all patients Intervention 2: repeated risk-assessment for pressure-injury prevention in high-risk groups Control: standard care	Model	CUA	Hospitalized adults	Societal and healthcare system perspective	USD (2017)	Costs of evaluating a patient for hospital-acquired pressure injuries, hospital-acquired pressure injuries, inpatient care, cost of prevention, and lost productivity	QALY	Societal perspective Intervention 1 vs. Control ICUR: $2000 (Int.$2503.78) Intervention 2 (Braden <15) vs. Control ICUR: dominant Intervention 2 (Braden <19) vs. Control ICUR: $622 (Int.$778.68) Health care perspective Intervention 1 vs. Control ICUR: $2142 (Int.$2681.55) Intervention 2 (Braden <15) vs. Control ICUR: dominant Intervention 2 (Braden <19) vs. Control ICUR: $622 (Int.$778.68)

Panneman et al., 2021 [52]	Netherlands	Intervention: multifactorial falls prevention Control: usual care	Model	CBA	Adult patients receiving chemotherapy	Healthcare system perspective	Euros (not stated, assumed 2019)	Intervention cost and cost of injury	Incremental net benefit and benefit-cost ratio	Incremental net benefit: €407 (Int.$620.89) Incremental benefit-cost ratio: 2.86

Silva et al., 2019 [50]	Brazil	Intervention: infusion pumps with drug library Control: conventional infusion pumps	Model	CEA	Patients in a pediatric Intensive Care unit	Not stated	BRL (2017)	Costs of hospitalization, equipment, and adverse events	Reduction of dose-related adverse events	ICER: R$4834.13 (Int.$3191.44)

Tan et al., 2016 [26]	China	Intervention: ultrasound-guided seldinger PICC Control: conventional method based on direct vein visualization	RCT	CEA	Adult patients	Not stated	CNY (not stated, assumed 2014)	Costs of catheterisation, maintenance and complication treatment	Catheterization effectiveness index	Cost Intervention: ¥3332.97 (Int.$1121.41) Control: ¥2855.38 (Int.$960.72) Catheterization effectiveness index Intervention: 89.29% Control: 59.18% ICER: ¥1591.97 (Int.$535.63)

Hälleberg et al., 2013 [24]	Sweden	Intervention: indwelling urinary catheterisation Control: intermittent catheterisation	RCT	CUA	Hip surgery patients	Not stated	Euros (2011)	Costs of material and labor, bladder scan, and urinary tract infection	QALY	Cost Intervention: €3954 (Int.$5723.05) Control: €3642 (Int.$5271.84) QALY Intervention: 0.093 Control: 0.090 ICUR: not reported

*Lower cost and lower effectiveness*										

Marsden et al., 2015 [41]	UK	Intervention: 4 hourly repositioning Control: alternating 2 and 4 hourly repositioning	Model	CUA	Older people	Healthcare system perspective	GBP (not stated, assumed 2013)	Costs of repositioning strategies and treating a pressure ulcer	QALY	Cost Intervention: £3656 (Int.$7080.38) Control: £4197 (Int.$8128.11) Incremental QALY: 0.000292 ICUR: £1 854 070 (Int.$3 590 678.91)

McFarland, 2017 [34]	UK	Intervention 1: pH paper testing of aspirate for determining nasogastric tube placement Intervention 2: Chest X-ray for determining nasogastric tube placement Control: no checking procedure	Model	CUA	Adult patients	Payer's perspective	GBP (not stated, assumed 2015)	Costs of nasogastric tube placement and confirmation and complications treatment	QALY	Cost Intervention1: £43.20 (Int.$82.02) Intervention2: £158.64 (Int.$301.21) Control: £0 (Int.$0) QALY Intervention 1: 0.11 Intervention 2: 0.12 Control: 0 ICUR (Intervention 1 vs. control): £392.73 (Int.$745.67) ICUR (Intervention 2 vs. Control): £1322.00 (Int.$2510.05)

Tuffaha et al., 2014 [25]	Australia	Intervention: clinically indicated catheter replacement strategy; control: routine replacement strategy	RCT	CEA/CBA	Adult patients	Healthcare system perspective	AUD (2010)	Costs of equipment and staff time	Rate of phlebitis avoided and incremental net monetary benefit	Cost Intervention: AUD61.70 (Int.$58.12) Control: AUD69.30 (Int.$65.28) Rate of phlebitis avoided Intervention: 92.84% Control: 93.25% Incremental net monetary benefit: AUD7.60 (Int.$7.16)

Wu et al., 2021 [27]	UK	Intervention 1: Hickman Intervention 2: PICC Intervention 3: PORTs	RCT	CUA/CEA	Adult patients	Healthcare system perspective	GBP (not stated, assumed 2019)	Costs of device, device insertion, device removal, device replacement, and complications	QALY, number of complications	Hickman vs. PICC Cost Hickman: £3262 (Int.$5748.78) PICCs: £1708 (Int.$3010.09) QALY Hickman: 0.763 PICCs: 0.755 Number of complications Hickman: 103 PICCs: 110 ICUR: £172 556 (Int.$304 103.84) PORTs vs. Hickman Cost PORTs: £2436 (Int.$4293.08) Hickman: £2481 (Int.$4372.39) QALY PORTs: 0.746 Hickman: 0.742 Number of complications PORTs: 73 Hickman: 131 ICUR: −£11 250 (–Int.$19 826.42) PORTs vs. PICCs Cost PORTs: £2706 (Int.$4768.92) PICCs: £1041 (Int.$1834.60) QALY PORTs: 0.741 PICCs: 0.759 Number of complications PORTs: 47 PICCs: 93 ICUR: –£56 (–Int.$98.69)

*Higher cost and lower effectiveness*										

Whitty et al., 2017 [42]	Australia	Intervention: patient-centred pressure ulcer prevention care bundle Control: standard care	Cluster RCT	CEA/CBA	Adult patients	Healthcare system perspective	AUD (2015)	Cost of intervention and preventive strategies	Probability of avoiding a HAPU, days free of HAPU, hospital length of stay, and incremental net monetary benefit	Cost Intervention: AUD243.91 (Int.$218.73) Control: AUD98.90 (Int.$88.69) Probability of avoiding a HAPU Intervention: 0.93 Control: 0.89 Days free of HAPU Intervention: 6.35 Control: 5.23 Hospital length of stay Intervention: 10.46 days Control: 7.78 days ICER : AUD3296 (Int.$2955.70) per HAPU case avoided ICER : AUD151 (Int.$135.41) per additional day free of HAPU Incremental net monetary benefit: –AUD2319.51 (–Int.$2080.03)

*Notes*. CUA: cost-utility analysis; CEA: cost-effectiveness analysis; CBA: cost-benefit analysis; CLABSI: central line-associated blood stream infection; HRTD: hydration response technology dressing; SAP: Zetuvit Plus (a super absorbent polymer dressing); SADM: DryMax Extra (a superabsorbent dressing); SAKM: Kerramax Care (superabsorbent dressing); SAE: Eclypse (superabsorbent dressing); Hickman: Hickman-type devices; PICC: peripherally inserted central catheters; PORTs: centrally inserted totally implantable venous access devices; HAPU: hospital-acquired pressure ulcer; ICER: incremental cost-effectiveness ratio; ICUR: incremental cost-utility ratio; USD: US dollars; AUD: Australian dollars; CNY: Chinese Yuan; GBP: Great Britain Pound £; BRL: Brazilian real R$; CAD: Canadian dollars; HKD: Hong Kong Dollar; JPY: Japanese Yen; DKK: Danish Krone; SEK: Swedish Krona.

## Data Availability

Data sharing is not applicable to this article as no new data were created or analyzed in this study.

## Supplementary Materials

Supplementary Materials Appendix 1: search strategies. Appendix 2: extended consensus on health economic criteria list (CHEC-list). Appendix 3: the studies excluded at full-text screening and reasons for exclusion. Appendix 4: results of risk of bias assessment using CHEC-extended checklist. Appendix 5: results of economic evaluations of clinical nursing practices. Appendix 6: dominant interventions identified from randomized controlled trials.
