# Supplementary material for: A Systematic Review of Economic Evaluations in Clinical Nursing Practices
Source: J Nurs Manag. 2024 Aug 9;2024:9939254. doi: 10.1155/2024/9939254 (PMC11918575; doi:10.1155/2024/9939254)
Supplement: Supplementary Materials — Appendix 1: search strategies. Appendix 2: extended consensus on health economic criteria list (CHEC-list). Appendix 3: the studies excluded at full-text screening and reasons for exclusion. Appendix 4: results of risk of bias assessment using CHEC-extended checklist. Appendix 5: results of economic evaluations of clinical nursing practices. Appendix 6: dominant interventions identified from randomized controlled trials. [file 9939254.f1.docx]

**Supplemental Materials**

**Appendix 1.** Search strategies.

**Appendix 2.** Extended Consensus on Health Economic Criteria list (CHEC-list).

**Appendix 3.** The studies excluded at full-text screening and reasons for exclusion.

**Appendix 4.** Results of risk of bias assessment using CHEC-extended checklist.

**Appendix 5.** Results of economic evaluations of clinical nursing practice.

**Appendix 6.** Dominant interventions identified from randomized controlled trials.

# Appendix 1. Search strategies

**Medline Via Ovid**

1 exp Cost-Benefit Analysis/

2 (economic evaluation* or evaluation*, economic).ti,ab.

3 (analys#s, cost benefit or analys#s, cost-benefit or "benefit? and cost?" or cost benefit or cost benefit analys#s or cost benefit data or "cost? and benefit?" or cost-benefit analys#s or cost-benefit data or data, cost-benefit).ti,ab.

4 (cost-utility or analysis, cost-utility or cost utility analysis or cost-utility analys#s).ti,ab.

5 (cost-effectiveness or cost effectiveness or cost effectiveness analysis or cost-effectiveness analysis or effectiveness, cost).ti,ab.

6 (analys#s, cost minimization or analys#s, cost-minimization or cost minimization analys#s or cost-minimization analys#s).ti,ab.

7 (analys#s, marginal or marginal analys#s).ti,ab.

8 exp Nurses/ or (nurse or nurse, registered or nurses or nurses, registered or nursing personnel or personnel, nursing or registered nurse or registered nurses).ti,ab.

9 exp Nursing Care/ or (care, nursing or nursing care).ti,ab.

10 exp Nursing Service, Hospital/ or (hospital nursing service* or nursing service*, hospital or service*, hospital nursing).ti,ab.

11 exp Nursing Services/ or (nursing service* or service*, nursing).ti,ab.

12 exp Nursing/ or Nurs$.ti,ab.

13 or/1-7

14 or/8-12

15 13 and 14

16 Animals/ not humans/

17 15 not 16

18 Historical article.pt.

19 Letter.pt.

20 Comment.pt.

21 Editorial.pt.

22 Systematic review.pt.

23 Review.pt.

24 Case Reports.pt.

25 or/18-24

26 17 not 25

27 limit 26 to english language

28 limit 27 to yr="2013 -Current"

Search date：23 January 2023

**Cochrane Central Register of Controlled Trials (CENTRAL) via Ovid**

1 exp Cost-Benefit Analysis/

2 (economic evaluation* or evaluation*, economic).ti,ab.

3 (analys#s, cost benefit or analys#s, cost-benefit or "benefit? and cost?" or cost benefit or cost benefit analys#s or cost benefit data or "cost? and benefit?" or cost-benefit analys#s or cost-benefit data or data, cost-benefit).ti,ab.

4 (cost-utility or analysis, cost-utility or cost utility analysis or cost-utility analys#s).ti,ab.

5 (cost-effectiveness or cost effectiveness or cost effectiveness analysis or cost-effectiveness analysis or effectiveness, cost).ti,ab.

6 (analys#s, cost minimization or analys#s, cost-minimization or cost minimization analys#s or cost-minimization analys#s).ti,ab.

7 (analys#s, marginal or marginal analys#s).ti,ab.

8 exp Nurses/ or (nurse or nurse, registered or nurses or nurses, registered or nursing personnel or personnel, nursing or registered nurse or registered nurses).ti,ab.

9 exp Nursing Care/ or (care, nursing or nursing care).ti,ab.

10 exp Nursing Service, Hospital/ or (hospital nursing service* or nursing service*, hospital or service*, hospital nursing).ti,ab.

11 exp Nursing Services/ or (nursing service* or service*, nursing).ti,ab.

12 exp Nursing/ or Nurs$.ti,ab.

13 or/1-7

14 or/8-12

15 13 and 14

16 Animals/ not humans/

17 15 not 16

18 Historical article.pt.

19 Letter.pt.

20 Comment.pt.

21 Editorial.pt.

22 Systematic review.pt.

23 Review.pt.

24 Case Reports.pt.

25 or/18-24

26 17 not 25

27 limit 26 to english language

28 limit 27 to yr="2013 -Current"

Search date：23 January 2023

**EMBASE**

1 'economic evaluation'/exp

2 'economic evaluation*':ab,ti

3 'cost benefit analysis'/exp

4 'cost benefit analys?s':ab,ti

5 'cost effectiveness analysis'/exp

6 'cost effectiveness analys?s':ab,ti

7 'cost utility analysis'/exp

8 'cost utility analys?s':ab,ti

9 'cost minimization analysis'/exp

10 'cost minimization analys?s':ab,ti

11 #1 OR #2 OR #3 OR #4 OR #5 OR #6 OR #7 OR #8 OR #9 OR #10

12 'nursing'/exp OR 'nurse'/exp

13 nurs*:ab,ti

14 #12 OR #13

15 #11 AND #14

16 #11 AND #14 AND [english]/lim

17 #11 AND #14 AND [english]/lim AND ([conference abstract]/lim OR [conference review]/lim OR [editorial]/lim OR [letter]/lim OR [review]/lim)

18 #16 NOT #17

19 'animal*' NOT 'human*'

20 #18 NOT #19

21 #18 NOT #19 AND [2013-2023]/py

Search date：23 January 2023

**CINAHL**

1. (MH "Cost Benefit Analysis")

2. AB economic evaluation* OR AB 'evaluation*, economic'

3. AB 'analys#s, cost benefit' or 'analys#s, cost-benefit' or 'benefit? and cost?' or 'cost benefit' or 'cost benefit analys#s' or 'cost benefit data' or 'cost? and benefit?' or 'cost-benefit analys#s' or 'cost-benefit data' or 'data, cost-benefit'

4. AB 'cost-utility' or 'analysis, cost-utility' or 'cost utility analys#s' or 'cost-utility analys#s'

5. AB 'cost-effectiveness' or 'cost effectiveness' or 'cost effectiveness analys#s' or 'cost-effectiveness analys#s' or 'effectiveness, cost'

6. AB 'analys#s, cost minimization' or 'analys#s, cost-minimization' or 'cost minimization analys#s' or 'cost-minimization analys#s'

7. AB 'analys#s, marginal' or 'marginal analys#s'

8. S1 OR S2 OR S3 OR S4 OR S5 OR S6 OR S7

9. AB Nursing Care OR AB nursing service* OR AB Nurs$ OR AB nurse* OR AB nursing

10. S8 AND S9

11. (MH "Animals+") NOT (MH "Human")

12. S10 NOT S11

13. S10 NOT S11 Limiters- Published Date: 20130101-current

14. S10 NOT S1 Limiters- Published Date: 20130101-current, Narrow by Language:- english

15. PT historical articles OR PT letters OR PT comments OR PT editorial OR PT (systematic review or meta-analysis) OR PT review OR PT case report

16. S14 NOT S15

Search date：23 January 2023

**NHS Economic Evaluation Database, Health Technology Assessment (CRD York)**

1. MeSH DESCRIPTOR Nursing Care EXPLODE ALL TREES IN NHSEED, FROM 2013 TO 2023

2. MeSH DESCRIPTOR Nursing Services EXPLODE ALL TREES IN NHSEED, FROM 2013 TO 2023

3. (nurs*) IN NHSEED, FROM 2013 TO 2023

4. 1 OR 2 OR 3

Search date：23 January 2023

**Web of science**

1 TS=("economic evaluation*" or "evaluation*, economic")

2 TS=("analys?s, cost benefit" or "analys?s, cost-benefit" or "benefit* and cost*" or "cost benefit" or "cost benefit analys?s" or "cost benefit data" or "cost* and benefit*" or "cost-benefit analys?s" or "cost-benefit data" or "data, cost-benefit")

3 TS=("cost-utility" or "analys?s, cost-utility" or "cost utility analys?s" or "cost-utility analys?s")

4 TS= ("cost-effectiveness" or "cost effectiveness" or "cost effectiveness analys?s" or "cost-effectiveness analys?s" or "effectiveness, cost")

5 TS= ("analys?s, cost minimization" or "analys?s, cost-minimization" or "cost minimization analys?s" or "cost-minimization analys?s")

6 TS=("analys?s, marginal" or "marginal analys?s")

7 TS=(nurs*)

8 #1 OR #2 OR #3 OR #4 OR #5 OR #6

9 #7 AND #8

10 #7 AND #8 and PY=(2013 or 2014 or 2015 or 2016 or 2017 or 2018 or 2019 or 2020 or 2021 or 2022 or 2023) and DT=Article and LA=English

Search date：23 January 2023

**Appendix 2.** Extended Consensus on Health Economic Criteria list (CHEC-list)^*^

| **Items** | **Assessment instructions** |
| --- | --- |
| 1.Is the study population clearly described? | The relevant clinical characteristics, entry and eligibility criteria, as well as drop-out during follow-up should be stated explicitly. |
| 2.Are competing alternatives clearly described? | A detailed description should be given of the competing interventions. This should encompass a clear and specific statement of the primary objective of each alternative, as well as relevant factors, such as intensity, duration, and frequency. |
| 3.Is a well-defined research question posed in answerable form? | A research question must identify clearly the alternatives being compared and the population for which the comparison is made. |
| 4.Is the economic study design appropriate to the stated objective? | An appropriate economic study design is a full economic evaluation (comparison of costs and effects of 2 or more interventions) based on primary research (cohort, case-control, randomised controlled trial). |
| 5.Are the structural assumptions and the validation methods of the model properly reported? | For models, the following information should be presented:  Structure:  -the structural hypotheses/ assumptions  -the uncertainty around these assumptions  -sources of information for these assumptions (systematic reviews preferred).  Validity:  Methods to verify the model’s  - structure (face) validity,  - performance (technical/internal) validity  - outcomes validity should be discussed. |
| 6.Is the chosen time horizon appropriate in order to include relevant costs and consequences? | The period of analysis of the study is the time horizon. This time horizon should always be equal for costs and outcomes if these are combined in a ratio. The time span should be long enough to include all relevant costs and outcomes relating the intervention. Ideally, the follow-up period should be extended till the situation is stabilised with reference to costs and effects. |
| 7.Is the actual perspective chosen appropriate? | ‘Perspective’ indicates from which point of view an economic evaluation study is performed. If the study is performed from a societal perspective tick ‘yes’, as all relevant costs and consequences of an interventions and disease are taken into account, if possible. Other narrower perspectives will only include certain components. The authors should motivate why a narrower perspective is valid. |
| 8.Are all important and relevant costs for each alternative identified? | A full identification of all important and relevant costs should be given in relation to the perspective and the research question. |
| 9.Are all costs measured appropriately in physical units? | The costs should be measured appropriately in physical units. The instrument by which the costs are measured should be valid and clearly stated (e.g. interview, questionnaire, cost-diary). |
| 10.Are costs valued appropriately? | The sources of valuation should be clearly stated for each cost price of every volume parameter and their reference year. The main cost should be calculated based on depleted sources, no tariffs should be used. |
| 11.Are all important and relevant outcomes for each alternative identified? | A full identification of all important and relevant outcomes should be given in relation to the perspective and the research question. |
| 12.Are all outcomes measured appropriately? | The outcome measurement should result from the outcome identification and this should be straightforward (e.g. if mortality is a main outcome measure this should be taken into account in the analysis). The instrument by which the outcomes are measured should be valid and clearly stated. |
| 13.Are outcomes valued appropriately? | The method of outcome valuation should be clearly stated. Examples of valuation methods are Discrete Choice Experiments (e.g. Conjoint analysis, Contingent valuation), Direct utility assessment (VAS, TTO, SG, etc.), Indirect utility assessment (HUI, EQ-5D, QWB, etc.), Person trade off, etc. |
| 14.Is an incremental analysis of costs and outcomes of alternatives performed? | An incremental analysis should examine the additional costs from one intervention over another, compared to the additional outcomes that it delivers. The incremental costs-effectiveness ratio is obtained by dividing the costs differences (C2-C1) by the outcome differences (O2-O1) for the alternatives. |
| 15.Are all future costs and outcomes discounted appropriately? | Discounting is done appropriately if all costs and outcomes are converted to one single year, based on a motivated discount rate. |
| 16.Are all important variables, whose values are uncertain, appropriately subjected to sensitivity analysis? | All variables in the analysis are potential candidates for the sensitivity analysis. Only variables that are certain or which have a minimal impact on the study results (based on the preliminary analysis) can be excluded from the sensitivity analysis. Furthermore, a justification should be given over the range of the variables used in the sensitivity analysis. |
| 17.Do the conclusions follow from the data reported? | Do the authors interpret their results cautiously and are their conclusions justified by the data. |
| 18.Does the study discuss the generalizability of the results to other settings and patient/client groups? | This can be done by being explicit about the viewpoint of analysis and by indicating how particular costs and outcomes vary by location, setting, patient population, care provider, etc. |
| 19.Does the article indicate that there is no potential conflict of interest of study researcher(s) and funder(s)? | If an external agency finances the study, a statement should explicitly be given about who finances the study to guarantee transparency in the relationship between the sponsor and the researcher. Whenever a potential conflict of interest is possible a declaration should be given of ‘competing interest’. |
| 20.Are ethical and distributional issues discussed appropriately? | Does the article notes ethical aspects and elaborates on the characteristics of the population experiencing the disease or the intervention (young, old, poor, wealthy) and how this may have distributional implications. |

* The checklist is from the publication: Odnoletkova I. CHEC-Extended: A tool for the quality assessment of economic evaluations of healthcare interventions. 2014.

# Appendix 3. The studies excluded at full-text screening and reasons for exclusion

| **Studies** | **Reason for exclusion** |
| --- | --- |
| Abdel-Fattah, M., Johnson, D., Constable, L., Thomas, R., Cotton, S., Tripathee, S., Cooper, D., Boran, S., Dimitropoulos, K., Evans, S., Granitsiotis, P., Hashim, H., Kilonzo, M., Larcombe, J., Little, P., MacLennan, S., Murchie, P., Myint, P.K., N'Dow, J., Norrie, J., Omar, M.I., Paterson, C., Scotland, G., Thiruchelvam, N., MacLennan, G., 2022. Randomised contolled trial comparing the clinical and cost-effectiveness of various washout policies versus no washout policy in preventing catheter associated complications in adults living with long-term catheters: study protocol for the CATHETER II study. Trials 23 (1), 630. | Not a full economic evaluation |
| Abu-Sheasha, G.A., Bedwani, R.N., Anwar, M.M., Yassine, O.G., 2020. Cost-effectiveness analysis of three methods of surgical-site infection surveillance: Less is more. Am J Infect Control 48 (10), 1220-1224. | Not target clinical nursing practices |
| Barton, G.R., Fairall, L., Bachmann, M.O., Uebel, K., Timmerman, V., Lombard, C., Zwarenstein, M., 2013. Cost-effectiveness of nurse-led versus doctor-led antiretroviral treatment in South Africa: pragmatic cluster randomised trial. Trop Med Int Health 18 (6), 769-777. | Not target clinical nursing practices |
| Blakely, T., Collinson, L., Kvizhinadze, G., Nair, N., Foster, R., Dennett, E., Sarfati, D., 2015. Cancer care coordinators in stage III colon cancer: a cost-utility analysis. BMC Health Serv Res 15 (1), 306-306. | Not target clinical nursing practices |
| Bleijenberg, N., Drubbel, I., Neslo, R.E.J., Schuurmans, M.J., ten Dam, V.H., Numans, M.E., de Wit, G.A., de Wit, N.J., 2017. Cost-Effectiveness of a Proactive Primary Care Program for Frail Older People: A Cluster-Randomized Controlled Trial. J Am Med Dir Assoc 18 (12), 1029-1036.e1023. | Not target clinical nursing practices |
| Blum, M.R., Øien, H., Carmichael, H.L., Heidenreich, P., Owens, D.K., Goldhaber-Fiebert, J.D., 2020. Cost-Effectiveness of Transitional Care Services After Hospitalization With Heart Failure. Ann Intern Med 172 (4), 248-257. | Not target clinical nursing practices |
| Bohingamu Mudiyanselage, S., Price, A.M.H., Mensah, F.K., Bryson, H.E., Perlen, S., Orsini, F., Hiscock, H., Dakin, P., Harris, D., Noble, K., Bruce, T., Kemp, L., Goldfeld, S., Gold, L., 2021. Economic evaluation of an Australian nurse home visiting programme: a randomised trial at 3 years. BMJ Open 11 (12), e052156. | Not target clinical nursing practices |
| Bruce, J., Hossain, A., Lall, R., Withers, E.J., Finnegan, S., Underwood, M., Ji, C., Bojke, C., Longo, R., Hulme, C., Hennings, S., Sheridan, R., Westacott, K., Ralhan, S., Martin, F., Davison, J., Shaw, F., Skelton, D.A., Treml, J., Willett, K., Lamb, S.E., 2021. Fall prevention interventions in primary care to reduce fractures and falls in people aged 70 years and over: the PreFIT three-arm cluster RCT. Health Technol Assess 25 (34), 1-114. | Not target clinical nursing practices |
| Campbell, J.L., Fletcher, E., Britten, N., Green, C., Holt, T., Lattimer, V., Richards, D.A., Richards, S.H., Salisbury, C., Taylor, R.S., Calitri, R., Bowyer, V., Chaplin, K., iyali, R., Murdoch, J., Price, L., Roscoe, J., Varley, A., Warren, F.C., 2015. The clinical effectiveness and cost-effectiveness of telephone triage for managing same-day consultation requests in general practice: a cluster randomised controlled trial comparing general practitioner-led and nurse-led management systems with usual care (the ESTEEM trial). Health Technol Assess 19 (42), 1-212. | Not target clinical nursing practices |
| Cartwright, C.C., Igbaseimokumo, U., Olsen, S., 2019. A Comparison of Dressing Techniques for Presurgical Closure of Myelomeningocele in the Neonate. J Neurosci Nurs 51 (5), 217-220. | Not a full economic evaluation |
| Chen, Y.-H., Lin, Y.-H., Hung, C.-S., Huang, C.-C., Yeih, D.-F., Chuang, P.-Y., Ho, Y.-L., Chen, M.-F., 2013. Clinical outcome and cost-effectiveness of a synchronous telehealth service for seniors and nonseniors with cardiovascular diseases: quasi-experimental study. J Med Internet Res 15 (4), e87-e87. | Not target clinical nursing practices |
| Choi Yoo, S.J., Nyman, J.A., Cheville, A.L., Kroenke, K., 2014. Cost effectiveness of telecare management for pain and depression in patients with cancer: Results from a randomized trial. Gen Hosp Psychiatry 36 (6), 599-606. | Not target clinical nursing practices |
| Cobos-Campos, R., Mar, J., Apiñaniz, A., de Lafuente, A.S., Parraza, N., Aizpuru, F., Orive, G., 2021. Cost-effectiveness analysis of text messaging to support health advice for smoking cessation. Cost Eff Resour Alloc 19 (1), 1-13. | Not target clinical nursing practices |
| Corbacho, B., Bell, K., Stamuli, E., Richardson, G., Ronaldson, S., Hood, K., ers, J., Robling, M., Torgerson, D., 2017. Cost-effectiveness of the Family Nurse Partnership (FNP) programme in England: Evidence from the building blocks trial. J Eval Clin Pract 23 (6), 1367-1374. | Not target clinical nursing practices |
| Driscoll, A., Gao, L., Watts, J.J., 2022. Clinical effectiveness and cost-effectiveness of ambulatory heart failure nurse-led services: an integrated review. BMC Cardiovasc Disord 22 (1), 1-11. | Not a full economic evaluation |
| Fealy, N., Osborne, C., Eastwood, G.M., Glassford, N., Hart, G., Bellomo, R., 2016. Nasal high-flow oxygen therapy in ICU: A before-and-after study. Aust Crit Care 29 (1), 17-22. | Not a full economic evaluation |
| Fletcher, J., Woodham, D., Cooper, S.C., 2021. Repair of central venous access devices in intestinal failure patients is safe and cost-effective: A retrospective single centre cohort study. Clin Nutr 40 (6), 4263-4266. | Not a full economic evaluation |
| Greving, J.P., Kaasjager, H.A.H., Vernooij, J.W.P., Hovens, M.M.C., Wierdsma, J., Gr, jean, H.M.H., Van Der Graaf, Y., De Wit, G.A., Visseren, F.L., 2015. Cost-effectiveness of a nurse-led internet-based vascular risk factor management programme: Economic evaluation alongside a randomised controlled clinical trial. BMJ Open 5 (5). | Not target clinical nursing practices |
| Grustam, A., Severens, J., De, M.D., Koymans, R., Vrijhoef, H., 2015. The cost-effectiveness analysis of philips motiva telehealth system: a comparison between home telemonitoring, nurse telephone support and usual care in chronic heart failure. Value Health 18 (7), 772-782. | Not target clinical nursing practices |
| Grustam, A.S., Severens, J.L., De Massari, D., Buyukkaramikli, N., Koymans, R., Vrijhoef, H.J.M., 2018. Cost-Effectiveness Analysis in Telehealth: A Comparison between Home Telemonitoring, Nurse Telephone Support, and Usual Care in Chronic Heart Failure Management. Value Health 21 (7), 772-782. | Not target clinical nursing practices |
| Grzeskowiak, L.E., Dekker, G., Rivers, K., Roberts-Thomson, K., Roy, A., Smith, B., Bowden, J., Bryce, R., Davies, M., Beilby, J., Wilson, A., Middleton, P., Ruffin, R., Karnon, J., Clifton, V.L., 2014. A randomized controlled trial to assess the clinical and cost effectiveness of a nurse-led Antenatal Asthma Management Service in South Australia (AAMS study). BMC Pregnancy Childbirth 14 (1). | Protocol |
| Guill, eacute, n-Sol, agrave, M., Soler, M.A., Tom, agrave, s-Vidal, A., Gaupp-Expert, P., 2013. A multi-center, randomized, clinical trial comparing adhesive polyurethane foam dressing and adhesive hydrocolloid dressing in patients with grade II pressure ulcers in primary care and nursing homes. BMC family practice 14 (196). | Protocol |
| Hernández, R.A., Jenkinson, D., Vale, L., Cuthbertson, B.H., 2014. Economic evaluation of nurse-led intensive care follow-up programmes compared with standard care: the PRaCTICaL trial. Eur J Health Econ 15 (3), 243-252. | Not target clinical nursing practices |
| Holtzer-Goor, K.M., Gaultney, J.G., Van Houten, P., Wagg, A.S., Huygens, S.A., Nielen, M.M., Albers-Heitner, C.P., Redekop, W.K., Rutten-Van Mölken, M.P., Al, M.J., 2015. Cost-effectiveness of including a nurse specialist in the treatment of urinary incontinence in primary care in the Netherlands. PLoS One 10 (10), e0138225. | Not target clinical nursing practices |
| Jinjing, W., Dean, K.S., Rosen, Z., Muennig, P.A., 2017. The Cost-effectiveness Analysis of Nurse-Family Partnership in the United States. J Health Care Poor Underserved 28 (4), 1578-1597. | Not target clinical nursing practices |
| Karr, J.C., de Mola, F.L., Pham, T., Tooke, L., 2013. Wound healing and cost-saving benefits of combining negative-pressure wound therapy with silver. Adv Skin Wound Care 26 (12), 562-565. | Not a full economic evaluation |
| Keeler, M., Haas, B.K., Northam, S., Nieswiadomy, M., McConnel, C., 2015. Analysis of costs and benefits of transparent, gauze, or no dressing for a tunnelled central venous catheter in Canadian stem cell transplant recipients. Canadian Oncology Nursing Journal 25 (3), 289-298. | Not a full economic evaluation |
| Khajehei, M., Gidaszewski, B., Maheshwari, R., McGee, T.M., 2022. Clinical outcomes and cost-effectiveness of large-scale midwifery-led, paediatrician-overseen home phototherapy and neonatal jaundice surveillance: A retrospective cohort study. J Paediatr Child Health 58 (7), 1159-1167. | Not target clinical nursing practices |
| Lemelin, A., Paré, G., Bernard, S., Godbout, A., 2020. Demonstrated Cost-Effectiveness of a Telehomecare Program for Gestational Diabetes Mellitus Management. Diabetes Technol Ther. 22 (3), 195-202. | Not target clinical nursing practices |
| Lettink, A., Chaibekava, K., Smits, L., Langenveld, J., van de Laar, R., Peeters, B., Verstappen, M.-L., Dirksen, C., Nieuwenhuijze, M., Scheepers, H., 2020. CCT: continuous care trial - a randomized controlled trial of the provision of continuous care during labor by maternity care assistants in the Netherlands. BMC Pregnancy & Childbirth 20 (1), N.PAG-N.PAG. | Protocol |
| Ling, R., Giles, M., Searles, A., 2021. Administration of indwelling urinary catheters in four Australian Hospitals: cost-effectiveness analysis of a multifaceted nurse-led intervention. BMC Health Services Research 21 (1), 1-11. | Not a full economic evaluation |
| Liu, J., Gormley, N., Dasenbrock, H.H., Aglio, L.S., Smith, T.R., Gormley, W.B., Robertson, F., 2019. Cost-Benefit Analysis of Transitional Care in Neurosurgery. Clinical Neurosurgery 85 (5), 672-679. | Not target clinical nursing practices |
| Livingston, T., Osborne, R., Botti, M., Chirgwin, J., Mihalopoulos, C., McGuigan, S., Heckel, L., Gunn, K., Ashley, D., Williams, M., Simons, K., 2013. Efficacy and cost-effectiveness of a telephone outcall program to reduce carer burden and depression among carers of cancer patients [protect]. Rationale and design of a multi-state, multi-centre randomised controlled trial. Asia Pacific journal of clinical oncology 9 (159), 2013-2011. | Protocol |
| Malakouti, S., Mirabzadeh, A., Nojomi, M., Ahmadi, T.A., Nadarkhani, F., Mirzaie, M., Chimeh, N., 2015. Clinical outcomes and cost effectiveness of two aftercare models provided by general physicians and nurses to patients with severe mental illness. Medical Journal of the Islamic Republic of Iran 29 (196). | Not target clinical nursing practices |
| Marek, K.D., Stetzer, F., Adams, S.J., Bub, L.D., Schlidt, A., Colorafi, K.J., 2014. Cost Utility Analysis of a Home-Based Nurse Care Coordination Program. J AM GERIATR SOC 62 (12), 83-96 | Not target clinical nursing practices |
| Martella, F., Salutari, V., Marchetti, C., Pisano, C., Di, N.M., Pietta, F., Centineo, D., Caringella, A., Musella, A., Fioretto, L., 2015. A retrospective analysis of trabectedin infusion by peripherally inserted central venous catheters: a multicentric Italian experience. Anti Cancer Drugs 26 (9), 990-994. | Not a full economic evaluation |
| Maru, S., Byrnes, J., Carrington, M.J., Chan, Y.-K., Stewart, S., Scuffham, P.A., 2018. Economic evaluation of a nurse-led home and clinic-based secondary prevention programme to prevent progressive cardiac dysfunction in high-risk individuals: The Nurse-led Intervention for Less Chronic Heart Failure (NIL-CHF) randomized controlled study. European Journal of Cardiovascular Nursing 17 (5), 439-445. | Not target clinical nursing practices |
| McPhail, J., 2019. Evaluating evidence for stoma care nursing: appraising a randomised controlled trial of ostomy skin barriers. Gastrointestinal Nursing 17 (7), 38-42. | Not a full economic evaluation |
| Menon, S., Thompson, L.-S., Tan, M., Chahal, S., Black, D., Hill, K.A., Gritton, D., Hathaway, C., Perry, I., 2016. Development and cost--benefit analysis of a nurse-led paracentesis and infusion service. Gastrointestinal Nursing 14 (9), 32-37. | Not target clinical nursing practices |
| Ndosi, M., Lewis, M., Hale, C., Bird, H., Ryan, S., Quinn, H., McIvor, E., Taylor, J., Burbage, G., Bond, D., White, J., Chagadama, D., Green, S., Kay, L., Pace, A., Bejarano, V., Emery, P., Hill, J., 2013. The cost-effectiveness of nurse-led care in people with RA. Rheumatology 52, 2013-2004. | Not target clinical nursing practices |
| Odnoletkova, I., Ramaekers, D., Nobels, F., Goderis, G., Aertgeerts, B., Annemans, L., 2016. Delivering diabetes education through nurse- led telecoaching. Cost-effectiveness analysis. PLoS One 11 (10). | Not target clinical nursing practices |
| Oksman, E., Linna, M., Hörhammer, I., Lammintakanen, J., Talja, M.P.-H.S., 2017. Cost-effectiveness analysis for a tele-based health coaching program for chronic disease in primary care. BMC Health Services Research 17, 1-7. | Not target clinical nursing practices |
| Painter, J.T., Fortney, J.C., Austen, M.A., Pyne, J.M., 2017. Cost-Effectiveness of Telemedicine-Based Collaborative Care for Posttraumatic Stress Disorder. Psychiatric Services 68 (11), 1157-1163. | Not target clinical nursing practices |
| Pozo-Martin, F., Akazili, J., Der, R., Laar, A., Adler, A.J., Lamptey, P., Griffiths, U.K., Vassall, A., 2021. Cost-effectiveness of a Community-based Hypertension Improvement Project (ComHIP) in Ghana: Results from a modelling study. BMJ Open 11 (9), e039594. | Not target clinical nursing practices |
| Rantz, M.J., Birtley, N.M., Flesner, M., Crecelius, C., Murray, C.S., 2017. Call to action: APRNs in U.S. nursing homes to improve care and reduce costs. Nursing Outlook 65 (6), 689-696. | Not a full economic evaluation |
| Reuben, D.B., Gill, T.M., Stevens, A., Williamson, J., Volpi, E., Lichtenstein, M., Jennings, L.A., Tan, Z., Evertson, L., Bass, D., Weitzman, L., Carnie, M., Wilson, N., Araujo, K., Charpentier, P., Meng, C., Greene, E.J., Dziura, J., Liu, J., Unger, E., Yang, M., Currie, K., Lenoir, K.M., Green, A.N.S., Abraham, S., Vernon, A., Samper-Ternent, R., Raji, M., Hirst, R.M., Galloway, R., Finney, G.R., Ladd, I., Rahm, A.K., Borek, P., Peduzzi, P., 2020. D-CARE: The Dementia Care Study: Design of a Pragmatic Trial of the Effectiveness and Cost Effectiveness of Health System–Based Versus Community-Based Dementia Care Versus Usual Dementia Care. Journal of the American Geriatrics Society 68 (11), 2492-2499. | Protocol |
| Richards‐Jones, S., Mihalopoulos, C., Heckel, L., Gunn, K.M., Tan, M., Livingston, P.M., Richards-Jones, S., 2019. An economic evaluation of a telephone outcall intervention for informal carers of cancer patients in Australia: An assessment of costs and quality-adjusted-life-years. Psycho-Oncology 28(3),525-532. | Not target clinical nursing practices |
| Ruschel, K., Souza, E., Mussi, C., Polanczyk, C., Clausell, N., Rabelo-Silva, E., 2013. Cost-effectiveness of home visit program for heart failure in a middle-income country. Journal of cardiac failure 19 (8). | Not target clinical nursing practices |
| Ruschel, K.B., Rabelo-Silva, E.R., Rohde, L.E., de Souza, E.N., Mussi, C.M., Polanczyk, C.A., 2018. Cost-Effectiveness of a Home Visit Program for Patients with Heart Failure in Brazil: Evidence from a Randomized Clinical Trial. Value in Health Regional Issues 17, 81-87. | Not target clinical nursing practices |
| Schumer, R.A., Guetschow, B.L., Ripoli, M.V., Phisitkul, P., Gardner, S.E., Femino, J.E., 2020. Preliminary Experience with Conservative Sharp Wound Debridement by Nurses in the Outpatient Management of Diabetic Foot Ulcers: Safety, Efficacy, and Economic Analysis. The Iowa orthopaedic journal 40 (1), 43-47. | Not a full economic evaluation |
| Sørensen, J., Primdahl, J., Horn, H.C., Hørslev-Petersen, K., 2015. Shared care or nurse consultations as an alternative to rheumatologist follow-up for rheumatoid arthritis (RA) outpatients with stable low disease-activity RA: cost-effectiveness based on a 2-year randomized trial. Scandinavian Journal of Rheumatology 44 (1), 13-21. | Not target clinical nursing practices |
| Sovi, N., Pajk, A., Jankowski, P., Duenas, A., Kawecka-Jaszcz, K., Wolfshaut-Wolak, R., Stepaniak, U., Kawalec, P.P., 2013. Cost-effectiveness of a cardiovascular disease primary prevention programme in a primary health care setting. Results of the Polish part of the EUROACTION project. Kardiologia Polska 71 (7), 702-711. | Not target clinical nursing practices |
| Van Dijk, S.E.M., Pols, A.D., Adriaanse, M.C., Bosmans, J.E., Elders, P.J.M., van Marwijk, H.W.J., van Tulder, M.W., 2013. Cost-effectiveness of a stepped-care intervention to prevent major depression in patients with type 2 diabetes mellitus and/or coronary heart disease and subthreshold depression: Design of a cluster-randomized controlled trial. BMC Psychiatry 13 (1), 128-138. | Protocol |
| Verweij, L., Petri, A.C.M., MacNeil-Vroomen, J.L., Jepma, P., Latour, C.H.M., Peters, R.J.G., op Reimer, W.J.M.S., Buurman, B.M., Bosmans, J.E.L., 2022. The Cardiac Care Bridge transitional care program for the management of older high risk cardiac patients: An economic evaluation alongside a randomized controlled trial. PLoS ONE 17 (1). | Not target clinical nursing practices |
| Walker, G., Todd, A., 2013. Nurse-led PICC insertion: is it cost effective? British Journal of Nursing 22 (19), S9-S15. | Not target clinical nursing practices |
| Walters, D., Gupta, A., Nam, A., Lake, J., Martino, F., Coyte, P., 2015. A Cost-Effectiveness Analysis of Low-Risk Deliveries: A Comparison of Midwives, Family Physicians and Obstetricians. Healthcare Policy 11 (1), 61-75. | Not target clinical nursing practices |
| Wang, J., Zou, X., Cong, L., Liu, H., 2018. Clinical effectiveness and cost-effectiveness of nurse-led care in Chinese patients with rheumatoid arthritis: A randomized trial comparing with rheumatologist-led care. International journal of nursing practice 24 (1). | Not target clinical nursing practices |
| Wright, W., Turner, S., Anopa, Y., McIntosh, E., Wu, O., Conway, D.I., Macpherson, L.M., McMahon, A.D., 2015. Comparison of the caries-protective effect of fluoride varnish with treatment as usual in nursery school attendees receiving preventive oral health support through the Childsmile oral health improvement programme - the Protecting Teeth@3 Study: a randomised controlled trial. BMC oral health 15, 160. | Protocol |
| Uchida, M., 2014. Comparative and Cost-Effectiveness Analyses of Resident Quality Outcomes in Nursing Homes. Columbia University, pp. 151 p-151 p. | Not a full economic evaluation |
| Youens, D., Parsons, R., Toye, C., Slatyer, S., Aoun, S., Hill, K.D., Skinner, M., Maher, S., Davis, S., Osseiran-Moisson, R., Moorin, R., 2019. The cost-effectiveness of a telephone-based intervention to support caregivers of older people discharged from hospital. BMC Geriatrics 19 (1), 1-11. | Not target clinical nursing practices |
| Zimmerman, L., Wilson, F., Schmaderer, M., Struwe, L., Pozehl, B., Paulman, A., Bratzke, L., Moore, K., Raetz, L., George, B., 2017. Cost-Effectiveness of a Care Transition Intervention Among Multimorbid Patients. Western Journal of Nursing Research 39 (5), 622-642. | Not target clinical nursing practices |

# Appendix 4. Results of risk of bias assessment using CHEC-extended checklist

**
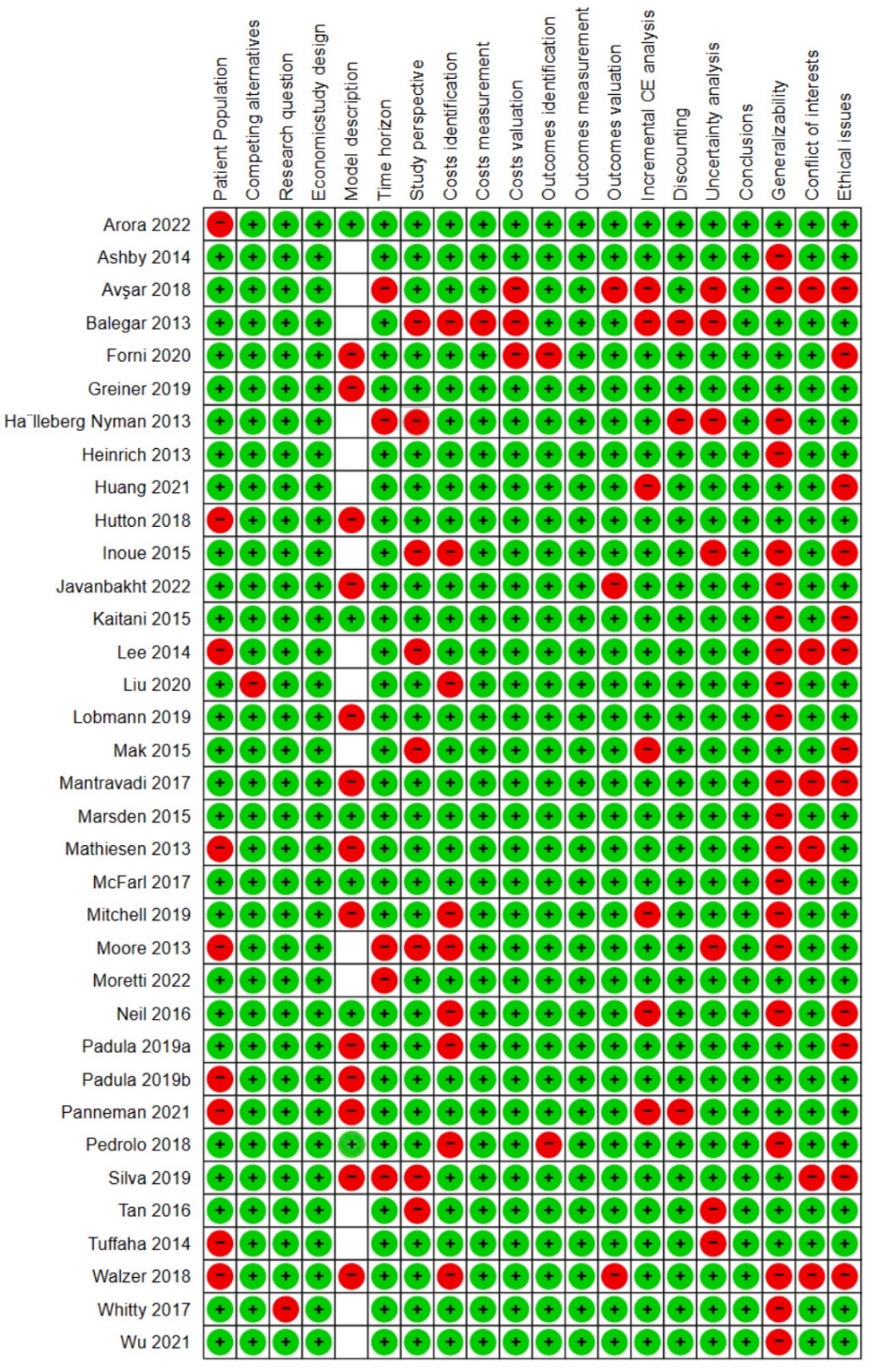
**

(Note: Green circle: low risk of bias, red circle: high risk of bias, empty box: not appliable)

**Appendix 5. Results of economic evaluations of clinical nursing practices**

**
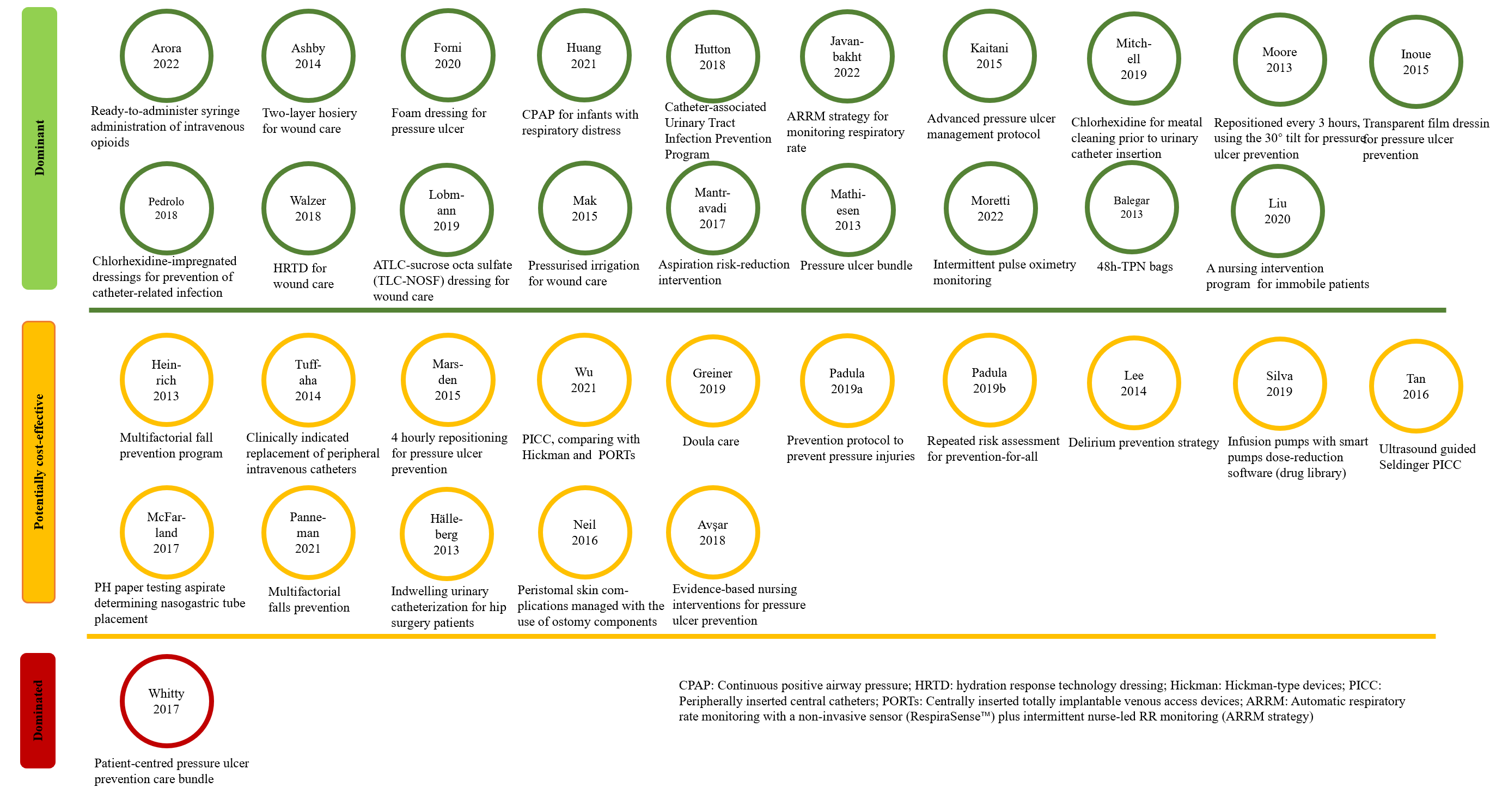
**

**Appendix 6.** Dominant interventions identified from randomized controlled trials

| **First author*, year** | **Country** | **Intervention and comparator** | **Evaluation type** | **Population** | **Perspective** | **Currency (price year)** | **Cost categories** | **Outcomes** | **Findings (costs in** **2024 int.$)** |
| --- | --- | --- | --- | --- | --- | --- | --- | --- | --- |
| Moore et al., 2013 [39] | Ireland | Intervention: Repositioned every 3 hours, using the 30° tilt;  Control: Repositioned every 6 hours, using the 90° lateral rotation | CEA | Older hospitalized patients | Not stated | Euros (not stated, assumed 2011) | Nurse time costs | Incidence of pressure ulcers | Cost:  Intervention: €206.6 (Int.$346.93)  Control: €253.1 (Int.$425.01)  Incidence of pressure ulcers:  Intervention: 3%  Control: 11%  ICER: dominant |
| Ashby et al., 2014 [20] | UK | Intervention: Two-layer hosiery;  Control: Four-layer bandage | CUA | Adults with venous leg ulcer | Societal perspective | GBP (not stated, assumed 2012) | Cost of trial compression treatments, cost of health-care consultations | QALY | Cost:  Intervention: £1492.9 (Int.$2953.97)  Control: £1795.3 (Int.$3552.32)  QALY:  Intervention: 0.685  Control: 0.651  ICUR: dominant |
| Moretti et al., 2022 [32] | Canada | Intervention: Intermittent pulse oximetry monitoring;  Control: Continuous pulse oximetry monitoring | CEA | Hospitalized infants with stabilized bronchiolitis | Societal and healthcare system perspective | CAD (2020) | Cost of hospital admission, cost of physician visits, cost of returning to emergency department and any hospital readmissions, cost of productivity lost, cost of childcare or caregiving | Length of hospital stays in hours | Cost:  *Societal perspective:*  Intervention: CAD6879 (Int.$6849.14)  Control: CAD7428 (Int.$7395.76)  *Health care system perspective:*  Intervention: CAD4195 (Int.$4176.79)  Control: CAD4716 (Int.$4695.53)  Length of stay:  Intervention: 37.4hours  Control: 38.5hours  ICER: dominant |
| Mak et al., 2015 [21] | China | Intervention: Pressurised irrigation;  Control: Swabbing method | CEA | Patients with wounds healing by secondary intention | Not stated | HKD (not stated, assumed 2013) | Cost of wound cleansing materials, cost of dressing fixation materials, cost of supplementary dressing materials, cost of nurse labour | Time-to-wound healing | Cost:  Intervention: HK$243.7 (Int.$52.72)  Control: HK$353.8 (Int.$76.53)  Time-to-wound healing:  Intervention: 11.4days  Control: 14.5days  ICER: dominant |
| Huang et al., 2021 [31] | Australia | Intervention: Continuous positive airway pressure (CPAP);  Control: Nasal high-flow (nHF) | CEA | Infants with respiratory distress | Healthcare system perspective | AUD (2019) | Inpatient costs at nontertiary special care nurseries, inpatient costs at tertiary NICU, costs of interhospital transfers | Intubation rate, NICU transfer rate | Cost:  Intervention: AUD20 606 (Int.$16 747.10)  Control: AUD 21 615 (Int.$17 567.14)  Intubation rate  Intervention: 5.9%  Control: 13.9%  NICU transfer rate  Intervention: 9.2%  Control:15.7%  ICER: dominant |

Notes: CUA: cost-utility analysis; CEA: cost-effectiveness analysis; CBA: cost-benefit analysis; ICER: Incremental cost-effectiveness ratio; ICUR: Incremental cost- utility ratio; AUD: Australia dollars; GBP: Great Britain Pound £; CAD: Canada dollars; HKD: Hong Kong Dollar. *Reference was shown in the main manuscript.
